# Inhibition of Quorum Sensing-Controlled Virulence Factors and Biofilm Formation in *Pseudomonas aeruginosa* by Culture Extract from Novel Bacterial Species of *Paenibacillus* Using a Rat Model of Chronic Lung Infection

**DOI:** 10.1155/2015/671562

**Published:** 2015-01-11

**Authors:** Saad Musbah Alasil, Rahmat Omar, Salmah Ismail, Mohd Yasim Yusof

**Affiliations:** ^1^Department of Microbiology, Faculty of Medicine, MAHSA University, 59100 Kuala Lumpur, Malaysia; ^2^Pantai Hospital Cheras, 56100 Kuala Lumpur, Malaysia; ^3^Institute of Biological Science, Faculty of Science, University of Malaya, 50603 Kuala Lumpur, Malaysia; ^4^Department of Medical Microbiology, Faculty of Medicine, University of Malaya, 50603 Kuala Lumpur, Malaysia

## Abstract

Quorum sensing (QS) is a key regulator of virulence factors and biofilm formation in Gram-negative bacteria such as *Pseudomonas aeruginosa*. Microorganisms that inhabit soil are of strategic importance in the discovery of compounds with anti-QS properties. The objective of the study was to test the culture extract of a taxonomically novel species of *Paenibacillus* strain 139SI for its inhibitory effects on the QS-controlled virulence factors and biofilm formation of *Pseudomonas aeruginosa* both *in vitro* and *in vivo*. The *Paenibacillus* sp. culture extract was used to test its anti-QS effects on the LasA protease, LasB elastase, pyoverdin production, and biofilm formation of *P. aeruginosa* as well as evaluate its therapeutic effects on lung bacteriology, pathology, hematological profile, and serum antibody responses of experimental animals in a rat model of chronic lung infection. Results showed significant decrease in the activities of QS-controlled LasA protease, LasB elastase pyoverdin, and biofilm formation of *P. aeruginosa* caused by the culture extract. Moreover, the extract significantly prolonged the survival times of rats and facilitated the clearance of biofilm infections from infected lungs. In conclusion, the antiquorum sensing effects of culture extract from a novel species of *Paenibacillus* provide new insights to combat biofilm-associated infections.

## 1. Introduction 

Quorum sensing (QS) is a key regulator of virulence factors and biofilm formation in Gram-negative bacteria such as* Pseudomonas aeruginosa* [1]. This QS system comprises a signal molecule, a synthetase to make this signal, and a regulator to regulate gene expression [2]. Several signaling molecules have been identified; however, the main molecules produced by Gram-negative bacteria are acylhomoserine lactones (AHLs) [3]. It has been reported that bacterial biofilms are associated with chronic infections such as cystic fibrosis (CF) and tonsillitis [4]. The discovery of QS system and its critical role in bacterial virulence has revealed new targets to attenuate their pathogenicity [2]. There are a number of ways to interrupt the QS system, one of which is the use of microbial natural products which represent an important step towards the discovery of novel therapeutic chemicals [5, 6]. Despite the fact that soil is arguably the most useful and valuable habitat on earth, it is still considered one of the least understood ecosystems that needs to be further explored [7]. Soil is a major source of bacteria that synthesize a wide range of compounds with versatile biological effects [8, 9]. An example of such microorganisms is the genus* Paenibacillus*. To date, there are 160 species of* Paenibacillus* approved and validated according to the bacterial nomenclature list by DSMZ [10]. These species produce a wide range of antibiotics [11]. Therefore, interest in* Paenibacillus* spp. as a source of new antimicrobial agents is increasing [12].

Advances in medical practice have led to the proper management of acute bacterial infections [13]. However, the efficiency of many antibiotics is currently decreasing due to the occurrence of multidrug resistant bacteria [14]. Pathogenic strains of* P. aeruginosa* possess the ability to form biofilms which contribute to its reduced susceptibility towards antibiotics and ability to cause chronic infections [2]. Since virulence factors and biofilm formation in Gram-negative bacteria are under the control of quorum sensing system, thus discovery of anti-QS compounds can be of great interest in the treatment of biofilm-associated chronic infections [2]. Moreover, the use of animal models is essential to gain a better understanding of the mechanisms involved in biofilm formation [15]. This approach is usually accomplished by infecting a vertebrate animal with the organism of choice followed by evaluation of the animal's immune responses [16]. In this study, culture extract from a taxonomically novel species of* Paenibacillus* isolated from an agricultural soil in Malaysia was tested for its QS inhibitory effects* in vitro* on LasA protease, LasB elastase, pyoverdin production, and biofilm formation of* P. aeruginosa* and evaluated for its antibiofilm therapeutic effects* in vivo* on lung bacteriology, lung pathology, hematological profile, and serum antibody responses* in vivo* using a rat model of chronic biofilm-associated lung infection.

## 2. Materials and Methods

### 2.1. Bacterial Isolates

*Paenibacillus* spp. are Gram-positive, facultatively aerobic, endospore-forming Bacilli. The strain 139SI (GenBank accession number: JF825470.1) from three strains of* Paenibacillus* isolates previously isolated from an agricultural soil in Malaysia was chosen as the type strain of the selected novel species. These strains were identified as members of the genus* Paenibacillus* on the basis of phenotypic characteristics, phylogenetic analysis, and 16S rRNA G+C content. The taxonomically novel species of* Paenibacillus* strain 139SI was deposited at the American Type Culture Collection (ATCC) with a cataloguing number (ATCC-BAA-2268) [17].

The strain was used to prepare the culture extract to examine its anti-QS inhibitory effects* in vitro* and* in vivo*. Upon approval from the medical ethics committee at University Malaya Medical Centre (UMMC) (PPUM/UPP/300/02/02 Ref. number 744.11), a clinical isolate of* Pseudomonas aeruginosa* was collected from the palatine tonsils of a patient undergoing elective tonsillectomy at UMMC. The isolate was identified via colony morphology, culturing on selective media and biochemical tests followed by assessment of its antibiotic susceptibility via disk diffusion where the isolate was shown to be multidrug resistant. The isolate was then used as the test strain in the preparation of test supernatant for LasA protease, LasB elastolytic, pyoverdin, and biofilm formation assays* in vitro* as well as the challenge strain in the rat model of chronic lung infection* in vivo*. Reference strain for the LasA assay included* Staphylococcus aureus *(ATCC 25923) whereas reference strains for the biofilm formation assay included* Pseudomonas aeruginosa* (ATCC 27853) and* Escherichia coli* (ATCC 25922) [18].

### 2.2. Chemicals

For the LasA protease, LasB elastolytic, pyoverdin, and biofilm formation assays as well as the rat model of chronic lung infection, commercially available anti-QS compound 2(5H)-Furanone 98% (Sigma-Aldrich) was used as the positive control. Furanones act by mimicking the AHL signal produced by Gram-negative bacteria, presumably by occupying the binding site on the putative regulatory protein, rendering it highly unstable and accelerating its turnover rate and thus resulting in the rapid disruption of the quorum sensing-mediated gene regulation [19].

For the rat model of chronic lung infection, commercially available sodium alginate powder (Sigma-Aldrich), that is, alginic acid sodium salt derived from brown algae, was used as the main component of the biofilm-alginate produced similarly by the live mucoid strains of* P. aeruginosa* to embed the challenge bacterial strain to closely resemble a lung infection caused by biofilms [20].

### 2.3. Preparation of* Paenibacillus* sp. Strain 139SI Culture Extract

In a previous study, the culture extract of* Paenibacillus *sp. strain 139SI was tested for its oral acute toxicity and was reported as nontoxic [21]. In our study, the extract will be tested for its QS inhibitory effect* in vitro* and therapeutic effects* in vivo*. A single colony from the novel* Paenibacillus *sp. strain 139SI was transferred into sterile brain heart infusion (BHI) broth (BD Difco) and incubated for 72 hours at 37°C to allow maximum secretion of bioactive secondary metabolites into the culture media. The culture was then transferred aseptically into 50 mL conical bottom centrifuge tube (Jet Bio Fil) and centrifuged at 8000 rpm for 20 min at 4°C to separate cell from supernatant. The obtained cell-free supernatant was then subjected to sterile filtration to remove unwanted particles using syringe filter with a pore size of 0.22 *μ*m (Minisart Sartorius) [22]. The sterile supernatant underwent freeze-drying (lyophilisation) and was then dissolved in ultra-pure water. The prepared stock was stored at −80°C to be used for* in vitro* and* in vivo* experiments.

### 2.4. Preparation of* Pseudomonas aeruginosa* Test Supernatant

For LasA protease, LasB elastolytic, and pyoverdin assays, a stationary-phase overnight culture of* Pseudomonas aeruginosa* clinical isolate was grown in LB medium at 37°C with shaking. The culture was then diluted 100-fold in LB medium and allowed to grow to an optical density at 600 nm (OD_600_). At this point, culture was divided into 10 mL aliquots and an additional volumes of 1 mL and 4.5 mL of* Paenibacillus *sp. culture extract was added to final concentrations of 1 mg/mL and 4.5 mg/mL [1].

### 2.5. LasA Protease Assay

The LasA staphylolyticprotease activity was measured by determining the ability of* P. aeruginosa* test supernatant to lyse boiled cells of* Staphylococcus aureus* [23]. A 30 mL volume of an overnight* S. aureus* culture was boiled for 10 min and then centrifuged for 10 min at 10,000 ×g (13,000 rpm). The resulting pellet was resuspended in 10 mM Na_2_PO_4_ (pH 4.5) to an OD_600_ of 0.5 McFarland standards. A 100 *μ*L aliquot of* P. aeruginosa* test supernatant with the* Paenibacillus *sp. culture extract was added to 900 *μ*L of the* S. aureus* suspension. Optical density of the mixture (OD_600_) was determined after 5, 10, 20, 30, 45, and 60 min. Negative control was LB medium alone whereas positive control was 2(5H)-Furanone. Activity was expressed as the change in OD_600_ per hour per *μ*g protein [1, 2].

### 2.6. LasB Elastolytic Assay

The elastolytic activity of the* P. aeruginosa* test supernatant was determined by using elastin Congo red (ECR) (Sigma-Aldrich) as described previously [24]. Briefly, a 100 *μ*L aliquot of test culture was added to 900 *μ*L of ECR buffer (100 mM Tris, 1 mM CaCl_2_, pH 7.5). The mixture was incubated with shaking for 3 hours at 37°C. Insoluble ECR was removed by centrifugation, and absorption of the* P. aeruginosa* test supernatant was measured at a wavelength of 495 nm. Negative control was LB medium alone whereas positive control was the 2(5H)-Furanone 98%. Activity was expressed as the change in the OD_495_ nm per *μ*g protein [1, 2].

### 2.7. Pyoverdin Assay

The pyoverdin assay was adapted from the methods of Cox and Adams [25]. Briefly, the prepared* P. aeruginosa* test supernatant was diluted 10-fold in Tris-HCl buffer (pH 7.4), and 100 *μ*L aliquots were added to 96-well microtiter plates on ice. Pyoverdin concentration was based on the fluorescence of the test supernatant at an excitation wavelength of 405 nm and an emission wavelength of 465 nm using a microtiter absorbance reader (iMark, Bio-Rad). Activity was expressed in relative fluorescence units (Table 1). Although pyoverdin is considered a marker of quorum sensing, a drop in its production may be due to an indirect effect via pH or iron concentration changes [26]. To eliminate the chance of false-positive results, the solution pH was checked throughout the experiment [1, 2].

### 2.8. Biofilm Formation Assay

The effect of* Paenibacillus *sp. strain 139SI culture extract on the attachment phase of biofilm formation was measured using microtiter plate (MTP) assay as described previously [27, 28]. Briefly, a 180 *μ*L aliquot of sterile LB broth and a 150 *μ*L aliquot of overnight culture of* P. aeruginosa* isolate were transferred into the 96-well microtiter plates. This was followed by adding a 150 *μ*L aliquot of* Paenibacillus *sp. culture extract with final concentrations of 1 mg/mL and 4.5 mg/mL and then incubated for 24 hours at 37°C. Wells were washed three times with phosphate-buffered saline to remove the weakly adherent cells and were allowed to air-dry prior to staining. The adherent biofilms were stained with a 200 *μ*L aliquot of 0.4% crystal violet solution (*w/v*) for 10 min. This was followed by adding a standard solution of 95% ethanol to extract (solubilize) the crystal violet from the stained biofilms. Optical density of biofilms was measured at a wavelength of 570 nm (OD_570_) using a microtiter absorbance reader (iMark, Bio-Rad) [29]. To compensate for possible differences in growth rates under different incubation conditions, the adherence index was adjusted as an estimate of the density of biofilm which would be generated by a culture with an OD_600_ of 0.5 McFarland standards [30]. Experiment was performed in triplicate and the data was then averaged and standard deviation was calculated. Calculation of the adherence index was done according to the following formula: adherence index = mean density of biofilm (OD_570_) × 0.5/mean growth (OD_600_).

### 2.9. Experimental Animals

The guidelines for care and use of laboratory animals were followed according to the National Academy of Sciences [31]. Experimental protocol was approved by the animal ethics committee at the Faculty of Medicine, University of Malaya (PM/27/07/2010/MAA (R)). A total of 48 adult Sprague Dawley (SD) rats including 24 males and 24 females were obtained from the animal care unit center (ACUC). Rats weighing 150–200 gm were kept in wire-bottomed cages at a 25°C temperature in a 12-hour light-dark cycle. Animals had free access to standard diet and water ad libitum. Body weight, clinical signs, and mortalities were observed and measured daily. Furthermore, general observations were recorded on the basis of behavioral changes such as food intake, salivation, muscular weakness, reflexes, and locomotion [32]. Rats were sacrificed under anesthesia with an intramuscular combination of Ketamine and Xylazine (1 mL of 100 mg/mL Xylazine + 9 mL of 100 mg/mL Ketamine) given at a dose of 0.1 mL/100 gm of body weight. On day 7 after infection, randomly selected rats from each group were sacrificed and their initial macroscopic and microscopic evaluation of acute inflammation was carried out. A longer observation period was chosen to allow lung abscess formation and a second examination was carried out on day 15 for all the surviving rats to evaluate their general observations and macroscopic and microscopic lung pathology.

### 2.10. Preparation of* Pseudomonas aeruginosa* Challenge Inoculation

The preparation of bacterial challenge inoculation was carried out as described previously [33]. Briefly,* P. aeruginosa* strain was cultured in 80 mL of BHI broth for 24 hours at 37°C with shaking (170 rpm). Cells were centrifuged for 30 min at ≥10,000 ×g (20,000 rpm) at 4°C and resuspended in 2 mL of fresh BHI broth and colony forming unit (cfu) was counted. To mimic the biofilm's environment,* P. aeruginosa* strain was immobilized in a solution of sodium alginate as described previously [34, 35]. Alginate powder was dissolved in normal saline to a concentration of 10 mg/mL and then autoclaved. A volume of 1 mL inoculation was mixed with 9 mL of sterile alginate. The mixture was stored at 4°C until being administrated intratracheally to experimental rats.

### 2.11. Experimental Design of Animal Model

The protocol to establish chronic lung infection was followed as described previously [36]. Rats were randomly divided into eight groups, with males in groups 1–4 and females in groups 5–8, as the following:

 negative control (groups 1 and 5), normal saline (5 mL/Kg, orally) daily + sterile distilled water (1 mL/Kg, orally) (daily for 14 days);
 positive control (groups 2 and 6), challenge bacterial inoculation (1 × 10^7^ cfu/rat) (5 mL/Kg, intratracheally) (twice at day 1 and 2 + sterile distilled water (1 mL/Kg, orally) daily for 14 days);
 comparative control (groups 3 and 7), challenge bacterial inoculation (1 × 10^7^ cfu/rat) (5 mL/Kg, intratracheally) (twice at day 1 and 2 + 2(5H)-Furanone (25 gm/Kg, orally) daily for 14 days);
 extract treatment (groups 4 and 8), challenge bacterial inoculation (1 × 10^7^ cfu/rat) (5 mL/Kg, intratracheally) (twice at day 1 and 2 +* Paenibacillus* sp. extract at concentration 4.5 mg/mL (25 gm/Kg, orally) daily for 14 days).

### 2.12. Evaluation of Lung Bacteriology

Upon sacrifice, the thoracic cavity of rats was opened by an excision through the peritoneum and lung specimens were harvested and cut into two parts. Unfixed lungs were prepared for quantitative bacteriological evaluation as described previously [14, 37]. Briefly, lungs were stored for 2 hours at 4°C and were mixed with 5 mL of cold sterile phosphate-buffered saline at 4°C. The mixture was then homogenized in a blender. Diluted homogenized samples were plated on nutrient agar plates to determine the bacterial colony forming unit (cfu) per lung after incubation for 24 hours at 37°C.

### 2.13. Lung Index of Macroscopic Pathology (LIMP)

The qualitative analysis of macroscopic lung pathology including abscess, consolidation, atelectasis, and hemorrhage was expressed as the lung index of macroscopic pathology (LIMP) which was calculated by dividing the area of left lung showing pathologic changes by the total area of the same lung [38]. Moreover, gross pathological changes in the lungs were assigned four different scores according to the severity of the inflammation as described previously [34, 39]: (I) normal lungs; (II) swollen lungs, hyperemia, and small atelectasis (≤10 mm^2^); (III) pleural adhesions and atelectasis (≤40 mm^2^); and (IV) abscesses, large atelectasis, and hemorrhages.

### 2.14. Microscopic Lung Pathology

Lung specimens were fixed with 10% neutral buffered formalin (NBF) for 24 hours and processed as described previously [40]. Lungs were embedded in paraffin wax using an embedding center (Leica EG1160, Leica Biosystems. Germany), sectioned using a microtome (Leica RM2135, Leica Biosystems. Germany) and fixed onto glass slides using a water bath (Leica HI1210, Leica Biosystems. Germany). Tissue sections were stained with Hematoxylin and Eosin (H&E) stain and mounted with diphenyl xylene (DPX) to be visualized using an upright light microscope (Eclipse LV150L, Nikon Instruments Inc., Japan). The examination was carried out as described previously [39]. Lung pathology was assigned microscopically one of four scores according to the severity of inflammation as follows: (1) normal histology; (2) mild focal inflammation; (3) moderate to severe focal inflammation with areas of normal tissue; and (4) severe inflammation to necrosis [38]. Lung cellular alterations were classified as acute or chronic inflammation [38, 39].

### 2.15. Microscopic Examination of Biofilms

Specimens were embedded in paraffin and were 5 *μ*m sections cut. Slides were stained for standard light microscopy using H&E and periodic acid Schiff (PAS) stains according to the manufacturers' instructions [41]. To preserve the biofilm's architecture, specimens were examined using confocal laser scanning microscope (CLSM) coupled with double fluorescent staining as described previously [42]. Tissue sections were embedded in optimal cutting temperature (OCT) media followed by snap freezing with a mixture of cold isopentane-liquid nitrogen to form solid blocks that were cut into a thickness of 5 *μ*m using a cryostat (Leica CM1850, Leica Microsystems. Germany). Each section was then fixed with 70% cold acetone for 10 min followed by double staining with 500 *μ*L propidium iodide (PI) for 5 min to detect bacterial cells in red followed by staining with 500 *μ*L Concanavalin A (Con A) fluorescent isothiocyanate (FITC) for 5 min to detect glycocalyx matrix in green. Sections were then washed in a solution of phosphate-buffered saline (PBS) and demineralized water and were embedded in a mounting medium of PBS/Glycerol containing an antiquenching agent (*p*-phenylenediamine).

Microscopic examination of stained sections was carried out using CLSM (LSM 700. Carl Zeiss, Germany) available at Universiti Putra Malaysia (UPM), Malaysia. The microscope was equipped with a krypton-argon laser for visualization of Con A FITC (number of signals acquired, 488 nm; emission 552 DF, 32 nm) and propidium iodide (number of signals acquired, 568 nm; emission 605 DF, 32 nm) [42]. Digital images of the optical sections were collected using ZEN 2010 software and converted to high-quality JPEG files using available software.

### 2.16. Differential Blood Count and Serum Immunoglobulins Level

Upon sacrifice, blood was drawn from the jugular vein under anesthesia. Collected blood specimens were immediately transported to the clinical diagnostic laboratories at University Malaya Medical Centre (UMMC). Whole blood was collected using the violet caped VACUETTE EDTA tubes for differential blood count test including neutrophils, lymphocytes, monocytes, eosinophils, and basophils.

Serum was collected using the red caped VACUETTE Serum tubes for immunoglobulins level in which their concentrations against* P*.* aeruginosa *standard antigen (St-Ag) were assessed by ELISA including serum antibody levels of IgG, IgA, IgM, and IgE [34]. ELISA units were obtained by dividing the mean absorbance of samples by the mean absorbance of internal standard expressing between 0 ± 30 and 0 ± 40 absorbance units [43].

### 2.17. Statistical Analysis

Statistical analysis was carried out using the Statistical Product and Service Solutions software (IBM SPSS statistics 21). Categorical data were compared by the *χ* ^2^ test, while unpaired differences in continuous data were compared by both the Mann-Whitney *U* test and the analysis of variance (ANOVA) test. All values were reported as standard error mean (S.E.M ±) and a probability value of (*P* < 0.05) was considered to be statistically significant.

## 3. Results

### 3.1. Inhibitory Effects on* P. aeruginosa* Virulence Factors by* Paenibacillus* sp. Extract

#### 3.1.1. Decreased Activity of LasA Protease and LasB Elastase

The LasA staphylolytic protease is a zinc metalloendopeptidase belonging to the *β*-lytic endopeptidase family of proteases [44]. There was a significant decrease in LasA activity compared to that of the control when* Pseudomonas aeruginosa* test supernatant was grown in the presence of* Paenibacillus *sp. culture extract at concentrations 1 mg/mL and 4.5 mg/mL. The LB medium negative control showed no significant change in LasA activity.

The LasB elastase is a zinc metalloprotease capable of destroying or inactivating a wide range of biological tissues and immunological agents [45]. There was a significant decrease in the OD readings due to the inhibitory effect by both* Paenibacillus* sp. culture extract and positive control 2(5H)-Furanone (Table 1).* Paenibacillus *sp. culture extract at a concentration of 4.5 mg/mL caused more decrease in the activities of both LasA protease and LasB elastase compared to concentration 1 mg/mL.

#### 3.1.2. Altered Production of Pyoverdin

Pyoverdins are virulence factors that compete with mammalian transferrin for iron [46] and promote pathogenicity by stimulating bacterial growth [25]. One of the pyoverdins is suggested to be a QS-like molecule, regulating both itself and the production of other toxins [47]. There was a significant decrease in the OD readings due to the inhibitory effect by both* Paenibacillus* sp. culture extract and positive control 2(5H)-Furanone (Table 1).* Paenibacillus *sp. culture extract at a concentration of 4.5 mg/mL exhibited decreased production of the QS-controlled pyoverdin compared to concentration 1 mg/mL. All of the tested cultures retained a pH of 7.0, regardless of the amount or the type of extract added.

#### 3.1.3. Decreased Biofilm Formation

Biofilm formation by* P. aeruginosa* leads to increased resistance and creates a severe infection in the lungs of patients with cystic fibrosis [48]. There was a significant decrease in the OD readings due to the inhibitory effect by both* Paenibacillus* sp. culture extract and positive control 2(5H)-Furanone (Table 1).* Paenibacillus *sp. culture extract at a concentration of 4.5 mg/mL caused decrease in the QS-controlled biofilm formation compared to concentration 1 mg/mL.

### 3.2. Effects of* Paenibacillus* sp. Extract on Experimental Rats

#### 3.2.1. Weight and Mortality

Oral administration of* Paenibacillus *sp. culture extract significantly prolonged the survival times of experimental rats in both treatment and comparative control groups. Insignificant mortalities occurred with 2 (16.6%) rats on days 3 and 7. Comparative control group had its share of mortalities with 3 (25%) rats on days 6 and 7. No mortalities were recorded among negative control group. All rats in positive control group died before completing the course of experiment; some died from a severe infection on day 3 after inoculation whereas others died on days 5 and 10. The greatest decrease of weight following administration of challenge* P. aeruginosa* strain was seen among positive control group followed by comparative then extract treatment groups.

#### 3.2.2. Clearance of Challenge Bacteria in the Lung

All rats, except for negative control group, were infected with a sublethal dose of challenge* P. aeruginosa* inoculation of 1 × 10^7^ cfu/rat (5 mL/Kg, intratracheally) thrice at days 1, 2, and 3. Immediately after inoculation, rats in comparative control group received 2(5H)-Furanone (25 gm/Kg, orally) daily for 14 days whereas rats in the treatment group received* Paenibacillus *sp. extract (25 gm/Kg, orally) daily for 14 days. On day 7 after infection, randomly selected rats from each group were sacrificed and their lung bacterial count was evaluated. The value of lung bacterial cfu in both comparative control and extract treatment groups dropped significantly on day 7 compared to those in positive control group. This means that the anti-QS effect of* Paenibacillus* sp. extract as well as the control compound 2(5H)-Furanone started to have its inhibitory effect within 7 days of their administration. Results suggested that infecting* P. aeruginosa* and their biofilms were cleared away more quickly in the groups receiving treatments with anti-QS compounds.

On day 15 after infection, all rats were sacrificed and the median of lung bacterial cfu in extract treatment group was found to be only one-fifth of that in negative control group and the difference was significant (*P* < 0.05), indicating that* Paenibacillus *sp. extract could influence the colonization and persistence of* P. aeruginosa* within the infected lungs. The value of lung bacterial cfu in positive control group is significantly higher than the values of the lung bacterial cfu in the rest of the groups (*P* < 0.05); this was due to colonization of* P. aeruginosa* in the lung without their QS system being interrupted. However, the values of lung bacterial cfu among comparative control group and extract treatment group did not differ and were statistically insignificant.

#### 3.2.3. Macroscopic Lung Pathology

In agreement with the results of lung bacteriology, milder macroscopic (gross) lung pathology was observed in both comparative control group and extract treatment group. On day 7 after infection, the macroscopic lung pathology of positive control group showed large haemorrhage and abscess (>40 mm^2^). A total of 2 rats died on days 3 and 5 after infection due to the severity of infection. However, the macroscopic lung pathology of comparative control group showed swollen lungs with hyperemia, small atelectasis, and moderate haemorrhage (10 mm^2^–40 mm^2^) whereas in extract treatment group the lungs were also swollen with hyperemia, atelectasis, and small haemorrhage (<10 mm^2^). Results indicated that* Paenibacillus *sp. culture extract significantly restricted the gross pathologic changes of lung to a smaller area during the acute phase of infection.

**Figure 1:**
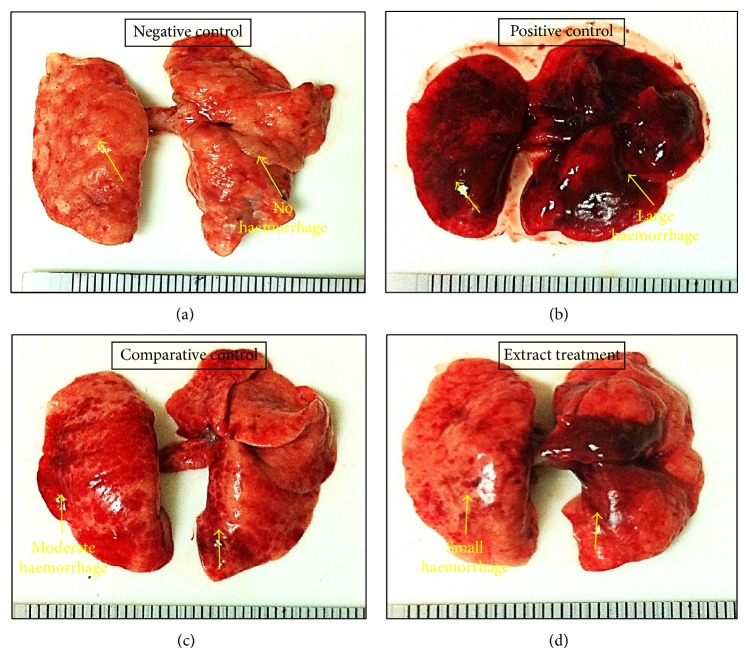
Macroscopic (gross) pathology of lungs among experimental groups based on the lung index of macroscopic pathology (LIMP). (a) Normal lung showing no signs of haemorrhage. (b) Infected lung with large haemorrhage and abscess (>40 mm^2^). (c) Swollen lungs with hyperemia, small atelectasis, and moderate haemorrhage (10 mm^2^–40 mm^2^). (d) Swollen lungs with hyperemia, atelectasis, and small haemorrhage (<10 mm^2^).

On day 15 after infection, lung abscesses and haemorrhage became predominant among positive control group. A significantly higher frequency of lung abscesses was noted in positive control group (*P* < 0.05) than in the comparative and extract treatment groups, in which 80% of rats showed lung atelectasis and only 20% of rats showed lung abscesses. The number of rats with chronic inflammation in both comparative control group 6 (12.5%) and extract treatment group 6 (12.5%) was significantly lower than that in positive control group 12 (25%). Intensity of lung infections on the gross pathology was more on day 15 after infection than day 7 in all groups except for negative control as there was no lung infection. Differences in gross pathology within each group between males and females were statistically insignificant. Results of lung index of macroscopic pathology are shown in (Figure 1).

#### 3.2.4. Microscopic Lung Pathology

**Figure 2:**
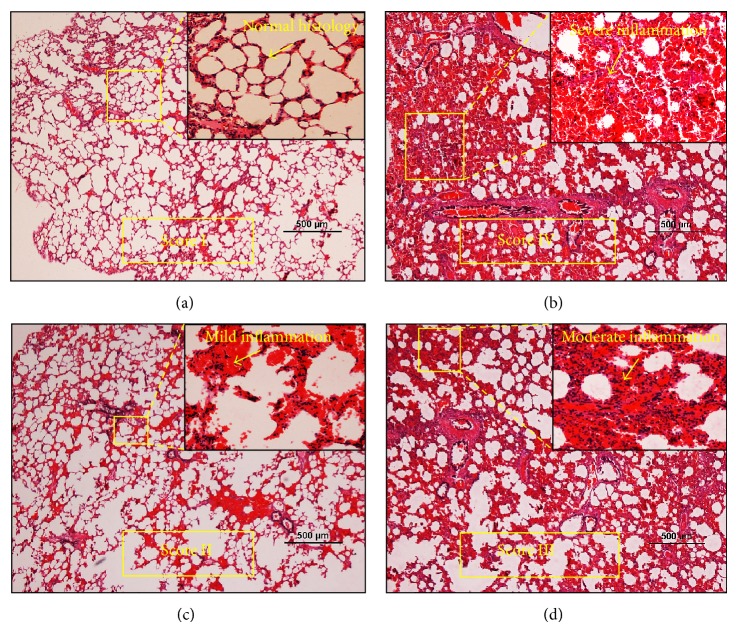
Histopathological scoring of lungs according to the severity of inflammation under low 4x and high magnifications 100x. (a) Score I with normal histology (negative control). (b) Score IV with severe inflammation to necrosis (positive control). (c) Score II with mild focal inflammation (extract treatment). (d) Score III with moderate to severe focal inflammation (comparative control). Arrows indicate the magnified areas of pathology scoring. Tissue sections stained with H&E stain.

On day 7 after infection, positive control group showed acute lung inflammation, multiple lung abscesses, haemorrhage, and consolidation whereas some rats died on days 3 and 5. Chronic inflammation was seen in all the 12 rats from positive control group. However, both comparative control and extract treatment groups showed chronic inflammation in 6 out of 12 rats whereas no inflammation was seen in negative control group. There were no differences in the inflammatory classification between comparative control and treatment groups. On day 15 after infection, the size of lung abscesses in comparative group was reduced compared to extract treatment group and the severity of chronic inflammation in general was decreased to half in these two groups. Pathology scoring showed normal lung tissue in all the rats, 12 (25%) of negative control group with Score I (Figure 2(a)), whereas 10 (20.83%) rats in positive control group showed severe inflammation to necrosis with Score IV (Figure 2(b)). Both comparative group 5 (10.4%) and extract treatment group 6 (12.5%) showed signs of recovery with Score II (Figure 2(c)) whereas 4 (8.33%) in comparative group and 5 (10.4%) in extract treatment group showed moderate to severe focal inflammation with Score III (Figure 2(d)). Differences in the inflammatory classification were insignificant between comparative control group 2 (4.16%) and extract treatment group 1 (2.08%) with Score IV (Figure 2(b)). Moreover, extract treatment group showed much milder chronic inflammation compared to comparative control where the areas with pathologic changes were smaller. The incidence of acute inflammation was lower than that in extract treatment group; however the differences were statistically insignificant. Results of microscopic lung pathology among experimental rats are shown in Table 2 and Figure 2.

#### 3.2.5. Disruption of Biofilms in the Lung

On day 7 after infection, sacrificed rats in positive control group showed dense biofilm layers occupying the lung alveolar spaces along with severe haemorrhage whereas some rats died on days 3 and 5 after inoculation with* P. aeruginosa*. Moreover on day 15 after infection, surviving rats among positive group were sacrificed and their lungs showed chronic inflammation with heavily dense biofilm layers blocking and occupying (80%) lung alveoli. In contrast, rats in both comparative group administered with the commercial anti-QS compound 2(5H)-Furanone and extract treatment groups treated with the* Paenibacillus *sp. culture extract showed milder inflammation with disrupted biofilm layers occupying (30%) lung alveoli. There was no significant difference in biofilm density within lung tissue between rats treated with 2(5H)-Furanone and those treated with the culture extract.

**Figure 3:**
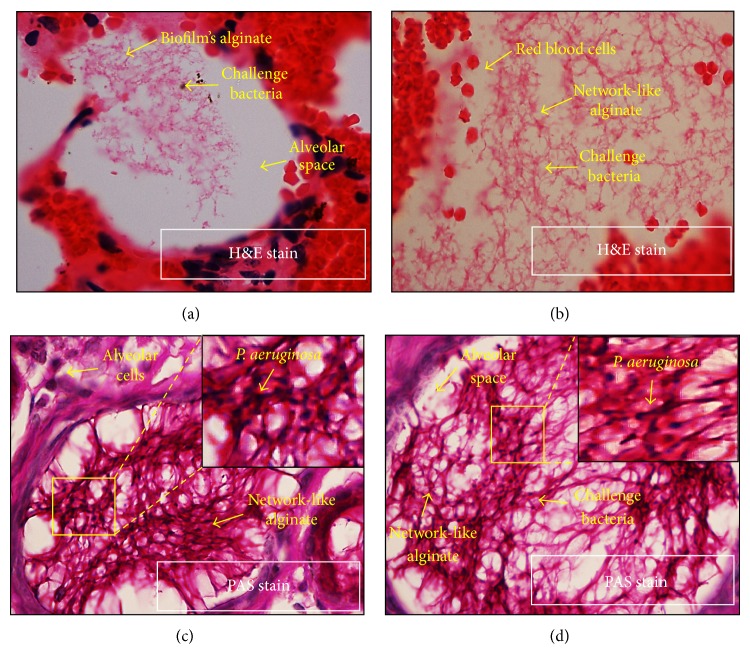
Microscopic evidence of representative biofilms of* P. aeruginosa* occupying alveolar spaces in different locations showing the pathogenic bacteria interconnected in a network-like structure of alginate (high magnification 100x); (a), (b) tissue sections stained with H&E stain; (c), (d) tissue sections stained with PAS stain.

**Figure 4:**
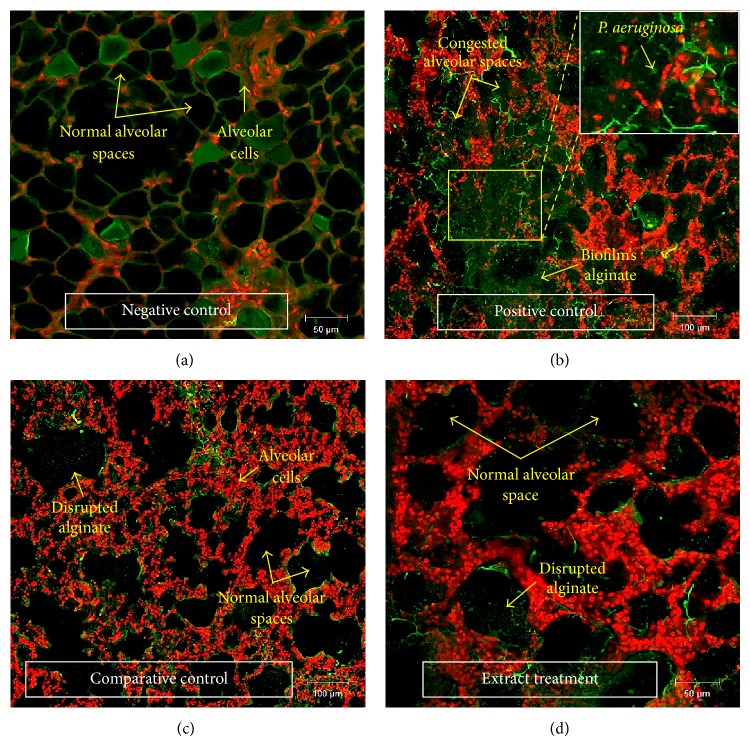
Microscopic examination of* P. aeruginosa* biofilms and their inflammation in lung tissues under confocal laser scanning microscope (CLSM). (a) Negative control group showing no biofilms with normal histology. (b) Positive control group showing intense biofilms filling the alveolar spaces with severe inflammation (high magnification 100x). (c) Comparative control group showing inhibited (disrupted) biofilms with mild inflammation. (d) Extract treatment group showing clearance of biofilms with mild inflammation. Arrows indicate the components of biofilms and lung alveoli. Tissue sections stained with propidium iodide (PI) and concanavalin A (Con A).

The challenge* P. aeruginosa* immobilized in seaweed alginate caused mechanical blocking and damage to the alveoli and was visualized under light microscope as microcolonies embedded in alginate by using H&E stain (Figures 3(a) and 3(b)) and PAS stain (Figures 3(c) and 3(d)). Moreover,* P. aeruginosa* was visualized under CLSM as interconnected bacteria (red) encased in a scaffolding network composed of extracellular matrix (green) by using double florescent staining (Figure 4). Under CLSM, microscopic examination revealed no biofilms with normal histology among rats of negative control group (Figure 4(a)) whereas positive control group showed intense biofilms filling the alveolar spaces with severe inflammation (Figure 4(b)). Both comparative control and extract treatment groups showed disrupted biofilms with mild inflammation (Figures 4(c) and 4(d)) indicating the same inhibitory effect of* Paenibacillus* sp. culture extract and control compound 2(5H)-Furanone in disrupting the* P. aeruginosa* biofilm. The intensity of biofilms occupying lung tissue was more on day 15 after infection than day 7 in both comparative control and extract treatment groups among males and females; however differences were statistically insignificant.

#### 3.2.6. Hematological Profile and Serum Antibody Responses

In agreement with the macroscopic and microscopic lung pathology, results of differential blood count (Table 3) and serum antibodies (Table 4) among negative control group showed normal levels and no significant differences (*P* > 0.05) due to an uncompromised immune status. In contrast, positive control group showed a significant increase in neutrophils, lymphocytes, and monocytes counts as well as an increase in IgM, IgG, and IgA levels indicating an immune response towards an infection.

On day 7 after infection, both comparative control and extract treatment groups showed a marked decrease in neutrophils, lymphocytes, and monocytes as well as IgM, IgG, and IgA levels compared to positive control group. This was due to the inhibitory effect of the administered* Paenibacillus* sp. culture extract on the infecting* P. aeruginosa* biofilms within lung alveoli. On day 15 after infection, positive control group showed a marked increase in differential blood count and immunoglobulins levels particularly IgM and IgG compared to comparative control and extract treatment group in which their IgM, IgG, and IgA levels were lower (*P* < 0.05). Eosinophils and basophils count IgE levels in all groups were within the reference normal range. Differences in differential blood account and serum antibody responses between male and female rats were statistically insignificant (*P* > 0.05).

## 4. Discussion

Among the promising approaches to combat biofilm infections is the use of metabolites synthesized from different bacterial species [49]. A large number of natural and synthetic compounds have been described exhibiting quorum sensing (QS) inhibitory effects against Gram-negative pathogens such as* Pseudomonas aeruginosa* [50]. Examples of such QS inhibitors are lactonase derived from* Bacillus* sp. [51], lactonase and acylase from actinobacteria and proteobacteria [52], Coral associated bacterial extracts [53], and* Bacillus* sp. SS4 that interferes with the AHL molecules [54]. These compounds function by either competing with the activity of AHL molecules due to their structural similarity or accelerating the degradation of the LuxR/LasR receptors of AHL. The virulence of* P. aeruginosa* is mainly due to its capacity to degrade host tissue with proteases and to form biofilms. Culture extract derived from a taxonomically novel species of* Paenibacillus* strain 139SI was tested for its ability to inhibit the QS-controlled virulence factors and biofilm formation of a multidrug resistant isolate of* P. aeruginosa in vitro* as well as its therapeutic effects* in vivo *using a rat model of chronic biofilm-associated lung infection. Since QS is involved in virulence factor production and biofilm formation of* P. aeruginosa* [55], we expected* Paenibacillus *sp. culture extract will have significant effects on the QS-controlled virulence factors. Indeed, the extract exhibited inhibitory effect on the ability of* P. aeruginosa* to produce virulence factors and biofilm formation. This was similar to the findings made by Adonizio et al. where they have detected anti-QS activities of six south Florida medicinal plants examined against* P. aeruginosa *PAO1 [1].

Disruption of the QS system with compounds such as halogenated Furanones from the Australian macroalgae* Delisea pulchra* [56] has also been shown to inhibit biofilm growth [57]. Therefore, in our study the compound 2(5H)-Furanone 98% was used as an anti-QS control for both* in vitro* and* in vivo* experiments showing a qualitative change in biofilm morphology and a reduction in its thickness. This was similar to the findings made by Hentzer et al. where they used Furanones from* D. pulchra *showing significant inhibition in biofilm formation [57].

### 4.1. Production of Virulence Factors

The LasA protease activity was determined by measuring the ability of* Paenibacillus* sp. culture extract to lyse boiled* Staphylococcus aureus* cells. There was a significant decrease in LasA activity compared to that of control when* P. aeruginosa* was grown in the presence of* Paenibacillus* sp. extract at a concentration of 4.5 mg/mL (75% decrease) compared to a concentration of 1 mg/mL (45% decrease) (Table 1). This was in agreement with a previous study by Adonizio et al. where they suggested that the compounds vescalagin and castalagin are responsible for the reduction in LasA activity [1]. There was a significant reduction in LasB elastase activity when* P. aeruginosa* was grown in the presence of culture extract at a concentration of 4.5 mg/mL (70% decrease) indicating that compounds in the tested extract may have downregulated the production of LasB and/or inhibited its activity. This was similar to the findings made by Rasmussen et al. where they reported a 50% decrease in LasB activity using 2% garlic [58]. Moreover, the control anti-QS compound, that is, 2(5H)-Furanone, showed a significant decrease (80%) in LasB activity similar to the findings made by Hentzer et al. where they reported a 90% decrease using purified halogenated Furanone from the red alga* Delisea pulchra* [59].

There was a significant reduction in pyoverdin production of* P. aeruginosa* with 65% reduction in pyoverdin levels by* Paenibacillus* sp. culture extract compared to the 2(5H)-Furanone which showed an 80% reduction. This was similar to the findings made by Hentzer et al. where they used synthetic derivate of natural Furanones with a 90% reduction in pyoverdin levels [59]. There were no significant changes in cell growth corresponding to pyoverdin production, leaving an anti-QS effect as the most likely hypothesis. However, further studies are needed to assess the effect of* Paenibacillus* sp. culture extract on direct enzyme inhibition of* P. aeruginosa* in comparison to quorum sensing signaling.

### 4.2. Biofilm Formation

Inhibition of biofilm formation without influencing bacterial growth is a characteristic of antivirulence therapies which are the promising alternatives to combat bacterial infections [60]. In our study, the use of* Paenibacillus* sp. culture extract at a concentration of 4.5 mg/mL resulted in a significant inhibition of biofilm formationin* P. aeruginosa*; however the influence of this extract on bacterial growth was insignificant. This study demonstrates that* Paenibacillus* sp. culture extract inhibits the QS-controlled biofilm formation of* P. aeruginosa* on hydrophobic surfaces (polystyrene) as indicated by the adherence index. This was similar to the previous findings suggesting that coated clinical materials with antimicrobial and anti-QS substances can lead to a successful prevention of microbial colonization [61, 62]. More studies on the active constituents of* Paenibacillus* sp. culture extract may be used as a tool to prevent microbial biofilm formation on hydrophobic and hydrophilic medicinal devices.

It has been reported that the QS gene expression in* P. aeruginosa* is interconnected with other regulatory systems that responds to various environmental signals [63]. The virulence factors LasA (protease) and LasB (elastase) are under the control of the* lasI-lasR* system [63]; however, the* rhlI-rhlR* system also controls activity to a lesser extent [64]. Pyoverdin is believed to be under the control of* rhlI-rhlR *[65], whereas biofilm formation is partially under the control of QS system [55]. Due to the redundancy of QS system in* P. aeruginosa* [66] and the complex chemistry of metabolites produced by* Paenibacillus *sp. culture extract, it was difficult to link the AHL level, QS gene expression, and virulence factor production. However, there was an overall inhibition of QS system in* P. aeruginosa* suggesting that multiple chemicals in the culture extract may have distinct QS inhibitory effects and that this effect is not directly on the* las-rhl* system but, rather, on a more universal QS regulator. This was similar to the findings made by Albus et al. where they have reported that the inhibitory effects of their compound maybe be due to a global QS regulator, such as Vfr [67] or GacA [68]. More studies are needed to identify the regulated QS genes, specificity of N-acyl-homoserine lactone signals, and environmental effects on gene expression.

### 4.3. Lung Bacteriology

Results of lung bacteriology showed that rats treated with both* Paenibacillus* sp. culture extract and control 2(5H)-Furanone exhibited lower bacterial numbers in lungs, indicating that the ability of challenge* P. aeruginosa* to colonize the lung may be reduced due to the inhibition of QS signals or because the bacterial clearance in the hosts is improved by the anti-QS effects of* Paenibacillus* sp. culture extract. Consequently, the inhibition of QS attenuates the virulence of* P. aeruginosa* and impairs its colonization ability. These results indicates that* Paenibacillus* sp. culture extract appears to be a promising novel antivirulent agent that possess quorum-sensing inhibition leading to increased clearance of bacteria from infected lungs and decreased lung pathology. This was similar to a recent proposal reporting that QS is a target for treatment of Gram-negative bacterial infections [69]. The effect of* Paenibacillus* sp. culture extract on bacterial clearance correlated negatively with their concentrations, which further confirmed that the action of Furanones was dosage-dependent. This was similar to the findings made by Wu et al. where they have reported synthetic Furanones to inhibit, in a dosage-dependent manner, the QS of* Pseudomonas aeruginosa* lung infection in mice [14].

### 4.4. Macroscopic and Microscopic Lung Pathology

Results showed that macroscopic lung pathology among extract treatment group was milder in comparison to positive control group. These findings suggest that the administered* Paenibacillus* sp. culture extract to* P. aeruginosa*-infected rats might induce an enhanced oxidative burst response that can aid in clearing the bacterial infection effectively and thus prolong survival time of rats with milder macroscopic lung pathology. This was similar to the findings made by Song et al. where they reported that the administration of ginseng extract leads to the activation of polymorphonuclear leukocyte (PMN) which reduced bacterial load in a rat model of chronic* P. aeruginosa* pneumonia. [38]. It is likely that the metabolites secreted by* Paenibacillus *sp. in the culture extract may have activated the endotoxin-primed neutrophils which resulted in reduced macroscopic and microscopic lung pathology. This was similar to a previous study where they have reported that Furanones successfully interfered with N-acyl homoserine lactone and suppressed bacterial QS which resulted in an accelerated clearance of* P. aeruginosa* in the lungs [14]. However, further studies are needed to address this aspect.

It has been reported that inhibition of the biofilm's alginate barrier using metabolites derived from culture extract of* Paenibacillus* sp. may be useful in the disruption of* P. aeruginosa* biofilms in infected lungs of cystic fibrosis patients [70]. In our study, it was noticed that the areas with chronic inflammatory changes in* Paenibacillus* sp. culture extract treatment group were smaller than that in positive control group. This was similar to a study by Song et al. where they have evaluated the effect of ginseng treatment on the oxidative burst response of peripheral blood neutrophils and alveolar macrophages in a rat model of chronic mucoid* P. aeruginosa* lung infection [38]. The fact that majority of rats classified under Score II belong to both comparative control group 5 (10.4%) and the treatment group 6 (12.5%) whereas majority under Score IV belong to the positive control group 10 (20.83%) indicates the anti-QS effects of* Paenibacillus* sp. culture extract in inhibiting the biofilm formation ability of* P. aeruginosa*.

### 4.5. Biofilm-Associated Lung Infection

In this study we have established a rat model of chronic lung infection based on* P. aeruginosa *isolate with the use of artificial embedding agents. Our animal experiment was accurate in developing the pulmonary infection since the number of bacteria cultured from lungs remained high throughout the infection period. Furthermore, the histopathological features in rats were similar to those of chronic* P. aeruginosa* lung infections in humans. This was similar to the findings made by Hoffmann et al. where they used a mucoid clinical isolate of* P. aeruginosa* NH57388A with hyperproduction of alginate in a mouse model of chronic lung infection [33]. The use of alginate to embed* P. aeruginosa* adds a selective survival advantage for the challenge bacterial strain. This was similar with a previous study where they reported improved persistence in mice lungs of mucoid* P. aeruginosa* strains relative to nonmucoid strains [71]. In contrast, another animal study comparing mucoid and nonmucoid* P. aeruginosa* strains failed to confer mucoidy as a selective advantage [72]. This discrepancy may reflect differences in the virulence of* P. aeruginosa* strains and/or the genetic background of the animals [73].

*P. aeruginosa* isolate embedded in alginate was able to establish a persistent lung infection in rats of all groups; however the severity of infection was lower in rats of both extract treatment and comparative control groups. This was probably due to the anti-QS effects of* Paenibacillus* sp. culture extract on the biofilm formation of* P. aeruginosa*. Since the genetic background of rats and the nature of biofilm influence the sensitivity of* P. aeruginosa* [73] we are not able to conclude that the differences are only due to the effect of* Paenibacillus *sp. culture extract.

*P. aeruginosa* secretes many proteases that play a major role in its pathogenesis [74]. These proteases such as elastase have been inversely correlated with alginate production [75]. The higher mortality seen in rat groups challenged with* P. aeruginosa* that were untreated with* Paenibacillus *sp. culture extract compared to treated ones may be due to the ability of untreated isolates to produce higher amounts of elastase and other QS-regulated virulence factors. This was similar to the study by Hoffmann et al. where they reported higher mortalities in mice challenged with the nonmucoid NH57388C isolate compared to the isogenic mucoid NH57388A isolate +QS [33].

Alginate from* P. aeruginosa* is a linear polymer of D-mannuronate and L-guluronate, which are highly soluble in water and chemically very similar to seaweed alginate [76]. Embedding* P. aeruginosa* with alginate solution enables the challenge strain to produce a more elastic biofilm structure as previously reported [77]. The anti-QS effects of* Paenibacillus* sp. culture extract caused weakness in the biofilm architecture and facilitated the clearance of its bacteria as visualized by both light microscope and confocal laser scanning microscope.

Histological examination of lung sections showed alveolar sacs invaded with* P. aeruginosa* biofilm anchored in a protective alginate matrix surrounded by numerous PMN. Despite this significant PMN infiltration, it did not appear to influence bacterial clearance. This finding supports previous* in vitro* studies showing that alginate protects bacteria from the host immune system [75]. The lungs of rats were characterized by foci of* P. aeruginosa* biofilms in the alveoli and alveolar ducts (Figures 4(a)–4(d)). This was similar to the findings by Tiddens where they have described the same pathological characteristics of those seen with CF patients [78]. However, differences in the therapeutic effects of* Paenibacillus *sp. culture extract on the histology of lungs in both extract treatment and comparative groups were insignificant suggesting the same mechanism of action based on inhibiting the QS system of* P. aeruginosa *and thus preventing its persistence via the formation biofilm.

### 4.6. Hematological Profile and Antibody Responses

Chronic* P*.* aeruginosa *lung infection in CF patients is characterized by a strong antibody response in the serum and infiltration of PMNs in the lung [79]. Our results showed high serum levels of IgM and IgG among positive control group than that in both comparative and treatment groups. The increased levels of serum antibodies could lead to increased levels of immune complexes in the lung foci which are thought to play an important role in the immunopathology of CF [43]. Results of hematological profile and serum antibody responses revealed that the challenge* P. aeruginosa *inhibited the immune response of all experimental rats, except negative control group, during the early acute phase of infection characterized by a significant increase count of neutrophils, lymphocytes, and monocytes but stimulated the humoral immune reaction during the chronic infection characterized by the marked increase of IgM, IgG, and IgE. Our results indicates that QS systems significantly affected the severity of* P*.* aeruginosa *lung infection in both acute and chronic phases and that the QS signals such as AHL in Gram-negative bacteria are promising new targets to combat virulence among patients with chronic* P*.* aeruginosa *lung infection. The activation of PMNs and downregulation of the IgG response might be associated with some changes in the production of cytokines. This was similar to the findings made by Song et al. where they have reported the association of ginseng treatment on neutrophil chemiluminescence and immunoglobulin G subclasses in a rat model of chronic* Pseudomonas aeruginosa* pneumonia [38]. Future studies are needed to investigate the role of* Paenibacillus* sp. culture extract and its identified compounds in the regulation of cytokine response using an animal model of biofilm-associated infection.

Our study shows that culture extract of* Paenibacillus* sp. can act as a potent antagonist of bacterial quorum sensing. Application of the extract and its potential compounds to Gram-negative biofilms, particularly* P. aeruginosa*, will increase bacterial susceptibility to antibiotics. Natural products such as microbial extract have long been a source of medicines [1]. However, most studies focus solely on bactericidal effects. Since the microbial culture extract in this study showed little, bactericidal activity, QS remains a potential mode of action. A shift of our focus to anti-QS and antivirulence properties within the bacterial community may reveal new anti-QS compounds and provide a novel method for the treatment of bacterial infections.

The* Paenibacillus* sp. culture extract along with the 2(5H)-Furanone control compound significantly increased the survival time of rats, assisted bacterial clearance, and reduced lung pathology, thus indicating that they could function as acyl-HSL antagonists and as quorum sensing inhibitors. Attenuation of bacterial virulence rather than killing the pathogen might become a new concept for control of bacterial infections [14]. This mode of action might not cause a selective pressure for the development of bacterial resistance to the antagonist.

In a previous study by Alasil et al., the molecular weight and chemical formula of three compounds from the* Paenibacillus* sp. culture extract were identified using ultra performance liquid chromatography-diode array detection (UPLC-DAD) and liquid chromatography-mass spectrometry (LC-MS) [21]. One of the reported compounds was a phospholipase A2 inhibitor with the name 4-hydroxy-5-(hydroxymethyl)-3-(14-methylpentadecanoyl) tetronicacid-2(5H)-Furanone with a similar chemical structure to the QS antagonist (5Z)-4-bromo-5-(bromomethylene)-3-butyl-2(5H)-Furanone from the algae* Delisea pulchra* inhibiting biofilm formation in* E. coli* without inhibiting its growth [80]. It is believed that the phospholipase A2 inhibitor compound in our* Paenibacillus* sp. culture extract could interfere with the QS signal acyl-HSL that may influence the type of host immune responses and reduce the inflammation* in vivo*. However, more studies are needed to test the acute toxicity of this phospholipase A2 inhibitor and assess its therapeutic effects* in vivo* on reducing the biofilm-associated infections among a larger sample size.

In conclusion, the effects of* Paenibacillus* sp. culture extract on* P. aeruginosa* are complicated and extend beyond the domain of QS-control hypothesis. However, the inhibition of virulence factors and biofilm formation provide insights into how natural product (metabolites) derived from a novel bacterial species of* Paenibacillus* can be used in the future to combat biofilm-associated infections caused by clinically important pathogens.

## Figures and Tables

**Table 1:** Quorum sensing inhibitory effects of *Paenibacillus* sp. culture extract on the virulence factors and biofilm formation of *Pseudomonas aeruginosa*.

Test (concentration)	Virulence factors			Biofilm formation	
	LasA protease activity^a^	LasB elastase activity^b^	Pyoverdin production^c^	Test clinical isolate *P. aeruginosa* ^d^	Control biofilm-forming isolate *P*. *aeruginosa* (ATCC 27853)^d^
	OD ± SD	OD ± SD	OD ± SD	OD ± SD	OD ± SD
With extract (1 mg/mL)	0.151 ± 0.013	88.333 ± 4.410	2261.000 ± 43.920	0.216 ± 0.073	0.124 ± 0.003
With extract (4.5 mg/mL)	0.091 ± 0.014	75.667 ± 8.090	2093.333 ± 95.614	0.149 ± 0.057	0.225 ± 0.004
Without extract (−control)	0.247 ± 0.004	167.000 ± 3.464	3775.667 ± 20.251	0.254 ± 0.003	0.539 ± 0.052
2(5H)-Furanone (+control)	0.062 ± 0.019	59.333 ± 9.244	1808.667 ± 139.190	0.105 ± 0.004	0.121 ± 0.001

^a^LasA activity is expressed as the reduction at OD_600_ per hour per μg of total protein.

^b^Elastase activity is expressed as the absorbance at OD_495_ per μg of protein.

^c^Pyoverdin production is expressed as the fluorescence at OD_465_ per μg of protein.

^d^Biofilm formation is expressed as the adherence index at OD_570_.

^d^OD > 0.24 = positive biofilm former isolate.

^d^OD > 0.12–<0.24 = weak biofilm former isolate.

^d^OD < 0.12 = negative biofilm former isolate.

Values are expressed as the standard error mean ± S.E.M.

Significant value is *P* < 0.05.

**Table 2:** Microscopic lung pathology among experimental rats.

Experimental groups (number of rats)	Microscopic lung pathology				
	Chronic inflammation Number (%)	Score I Number (%)	Score II Number (%)	Score III Number (%)	Score IV Number (%)
Negative control (12)	0	12 (25%)	0	0	0
Positive control (12)	12 (25%)	0	0	2 (4.16%)	10 (20.83%)
Comparative control (12)	6 (12.5%)	1 (2.08%)	5 (10.4%)	4 (8.33%)	2 (4.16%)
Extract treatment (12)	6 (12.5%)	0	6 (12.5%)	5 (10.4%)	1 (2.08%)
Total (48)	**18** (**37.5%**)	**13** (**27.08%**)	**11** (**22.91%**)	**11** (**22.91%**)	**13** (**27.08%**)

**Table 3:** Differential blood count among male and female experimental rats.

Differential blood count	Experimental groups				Reference range
	Negative control	Positive control	Comparative control	Extract treatment	
Neutrophils	4.4167 ± 0.39807	9.7500 ± 0.32856^*^	7.6667 ± 0.33333^*^	7.9167 ± 0.25990^*^	2.00–7.00 (10^9^/L)
Lymphocytes	2.0833 ± 0.19300	5.6667 ± 0.37605^*^	3.5000 ± 0.31382^*^	3.7500 ± 0.27866^*^	1.00–3.00 (10^9^/L)
Monocytes	0.5175 ± 0.16522	2.0225 ± 0.13644^*^	1.1583 ± 0.11808^*^	1.1342 ± 0.07834^*^	0.20–1.00 (10^9^/L)
Eosinophils	0.2442 ± 0.03506	1.0167 ± 0.06723^*^	0.5117 ± 0.04489^*^	0.5083 ± 0.03362	0.02–0.50 (10^9^/L)
Basophils	0.0442 ± 0.00514	0.1675 ± 0.00605	0.0992 ± 0.00583	0.1008 ± 0.00358	0.02–0.10 (10^9^/L)

Values are expressed as the standard error mean ± S.E.M.

^*^Values that are above or below the control reference range. Significant value is *P* < 0.05.

**Table 4:** Serum immunoglobulins level among male and female experimental rats.

Immunoglobulins	Experimental groups				Reference range
	Negative control	Positive control	Comparative control	Extract treatment	
IgM	145.6667 ± 13.76113	542.0833 ± 22.03663^*^	164.2500 ± 24.09109	231.9167 ± 13.77110	34–265 mg/dL
IgG	988.4167 ± 36.81618	2364.7500 ± 57.47004^*^	1891.9167 ± 73.15327	1856.4167 ± 70.50257	931–1916 mg/dL
IgA	136.0833 ± 10.67457	531.2500 ± 16.55300^*^	295.6667 ± 27.50932	360.4167 ± 29.22262	70–473 mg/dL
IgE	42.8333 ± 3.55867	120.7500 ± 8.99758	109.5000 ± 5.54254	112.7500 ± 4.85724	<165 IU/mL

Values are expressed as the standard error mean ± S.E.M.

^*^Values that are above or below the control reference range. Significant value is *P* < 0.05.

IU/mL = international unit per milliliter, mg/dL = milligram per decilitre.
